# Potential of immune-related genes as promising biomarkers for premature coronary heart disease through high throughput sequencing and integrated bioinformatics analysis

**DOI:** 10.3389/fcvm.2022.893502

**Published:** 2022-08-26

**Authors:** Haiming Wang, Junjie Shao, Xuechun Lu, Min Jiang, Xin Li, Zifan Liu, Yunzhang Zhao, Jingjing Zhou, Lejian Lin, Lin Wang, Qiang Xu, Yundai Chen, Ran Zhang

**Affiliations:** ^1^Department of Cardiovascular Medicine, Chinese PLA General Hospital and Chinese People's Liberation Army (PLA) Medical School, Beijing, China; ^2^Department of Hematology, The Second Medical Center of Chinese PLA General Hospital and Chinese People's Liberation Army (PLA) Medical School, Beijing, China; ^3^Department of Respiratory and Critical Care, Chinese People's Liberation Army (PLA) General Hospital, Beijing, China; ^4^Department of Health Services, The First Medical Center of Chinese People's Liberation Army (PLA) General Hospital, Beijing, China

**Keywords:** premature coronary heart disease, high throughput sequencing, integrated bioinformatics analysis, immune dysfunction, potential biomarkers

## Abstract

**Background:**

Coronary heart disease (CHD) is the most common progressive disease that is difficult to diagnose and predict in the young asymptomatic period. Our study explored a mechanistic understanding of the genetic effects of premature CHD (PCHD) and provided potential biomarkers and treatment targets for further research through high throughput sequencing and integrated bioinformatics analysis.

**Methods:**

High throughput sequencing was performed among recruited patients with PCHD and young healthy individuals, and CHD-related microarray datasets were obtained from the Gene Expression Omnibus (GEO) database. Differentially expressed genes (DEGs) were identified by using R software. Enrichment analysis and CIBERSORT were performed to explore the enriched pathways of DEGs and the characteristics of infiltrating immune cells. Hub genes identified by protein–protein interaction (PPI) networks were used to construct the competitive endogenous RNA (ceRNA) networks. Potential drugs were predicted by using the Drug Gene Interaction Database (DGIdb).

**Results:**

A total of 35 DEGs were identified from the sequencing dataset and GEO database by the Venn Diagram. Enrichment analysis indicated that DEGs are mostly enriched in excessive immune activation pathways and signal transduction. CIBERSORT exhibited that resting memory CD4 T cells and neutrophils were more abundant, and M2 macrophages, CD8 T cells, and naïve CD4 T cells were relatively scarce in patients with PCHD. After the identification of 10 hub gens, three ceRNA networks of *CD83, CXCL8*, and *NR4A2* were constructed by data retrieval and validation. In addition, *CXCL8* might interact most with multiple chemical compounds mainly consisting of anti-inflammatory drugs.

**Conclusions:**

The immune dysfunction mainly contributes to the pathogenesis of PCHD, and three ceRNA networks of *CD83, CXCL8*, and *NR4A2* may be potential candidate biomarkers for early diagnosis and treatment targets of PCHD.

## Introduction

Coronary heart disease (CHD) is associated with high morbidity, mortality, and economic burdens, and it has emerged as a leading health problem worldwide that is generally caused by multiple cardiovascular risk factors, including diabetes, hyperlipidemia, hypertension, cigarette smoking, and an unhealthy lifestyle (1). With recent substantial progress in exploring the progression of coronary atherosclerosis, classic cardiovascular risk factors are inadequate as indicators of disease prediction and stratification (2), and inflammatory biomarkers such as C-reactive protein (CRP) and interleukin-6 (IL-6) have received growing attention in the pathogenesis of CHD (3, 4). The currently available therapeutic strategies in clinical practice have not yet completely prevented or reversed coronary artery damage (5, 6), and this disease occurs increasingly at a younger age (7). Premature CHD (PCHD) is considered an acute myocardial infarction, with more than 70% stenosis of the coronary arteries observed in coronary angiography before the age of 45 (7). The accumulating evidence has found that approximately 40% of predispositions for patients with CHD are inherited, and genetic variation is therefore considered a non-negligible risk factor for these special populations (2, 8). The identification of potential molecular mechanisms and novel detectable biomarkers is essential for early clinical diagnosis, individualized medication, and improved prognosis of CHD (9).

Currently, the extensive application of high throughput sequencing, microarray profiling, and integrated bioinformatics analysis made a breakthrough in the discovery of abundant differentially expressed genes (DEGs) that participate in various diseases (9, 10). These DEGs show close relationships to their multiple biological functions of CHD, and these technologies can therefore provide insight into the deleterious mechanisms of CHD from the genome-wide dimension (9, 11, 12). The detection and construction of competitive endogenous RNA (ceRNA) networks based on DEGs could elucidate a transcriptional regulatory mechanism in disease progression and recovery in detail (13). Additionally, these circulating DEGs are viewed as promising candidate biomarkers because they are relatively stable, easily detectable, and a result of their system-specific attributes (11). In this study, we recruited 45 Chinese Han patients for PCHD, combined sequencing, and bioinformatics analysis to make comparisons of whole blood transcriptome differences in individuals with and without PCHD, and identified potential targets for early diagnosis, stratification, and intervention.

## Materials and methods

### Participant recruitment and sample collection

This study was approved by the ethics committee of the Chinese PLA General Hospital, and only young adults under the age of 45 were recruited. A total of 45 patients with PCHD were eligible for enrollment based on the results of diagnostic coronary angiography. We excluded the patients with aortic dissection, pulmonary embolism, cardiomyopathy, valvular heart disease, and a history of previous cancer, current infection, autoimmune diseases, or hemopathy. Another population encompassed eight healthy individuals who were enrolled as the control group. All participants with informed consent provided fasting blood samples for high throughput sequencing the next morning after admission to exclude physiological interfering factors, such as eating, fasting, and diurnal rhythms.

### Total RNAs extraction, sequencing, and data processing

The red blood cells were lysed and total RNAs were isolated from whole blood samples by using TRIzol^®^ reagent (Wuhan ServiceBio Technology, Wuhan, China) following the manufacturer's instructions. We used 1% agarose gel electrophoresis to evaluate the degradation and contamination of RNA. The RNA purity and concentration were calculated *via* a NanoDrop 2000 spectrophotometer (Thermo Scientific, MA, USA). The RNA integrity was accurately assessed by using the Agilent 2100 Bioanalyzer (Agilent Technologies, Santa Clara, CA, USA). Then, RNA sequencing libraries were constructed by NEBNext^®^ Ultra™ Directional RNA Library Prep Kit for Illumina^®^ (NEB, Ispawich, USA). Given that some rRNA and circulating non-coding RNAs will result in data bias in RNA-seq technology, the rRNA and circulating non-coding RNAs have been eliminated by the kit when the library was established. To further reduce the influence of these RNAs, we would also screen and eliminate rRNAs based on the quality control results of fastqc to ensure the accuracy of the final quantitative results of each gene in the comparison analysis. Libraries were then tested for quality *via* the Agilent 2100 Bioanalyzer, quantified using qPCR (Kapa Biosystems, Woburn, MA, USA), and sequenced on an Illumina HiSeq™ 2000 sequencing platform (Illumina, San Diego, CA, USA), following the manufacturer's protocols. We filtered raw sequencing reads *via* the following three criteria to ensure the quality of information analysis: (1) paired reads were discarded if the percentage of unknown bases was more than 10% in single-ended reads; (2) paired reads with sequencing joints or adaptors were discarded; and (3) paired reads were discarded if the percentage of low-quality bases was over 50% in single-ended reads. In addition, the Q20, Q30, and GC contents of all sequencing reads were detected and thus high-quality clean reads were selected for subsequent analysis.

The genome reference files and gene model annotations were downloaded from the genome website. The reference genome index was generated *via* Bowtie2 software and then paired-end clean reads were aligned with the reference genome based on HISA T2 software (14). Cufflinks 2.0 program was applied to assemble separately the transcriptome of each sample. We used Cuffmerge to combine all transcriptomes to produce a final transcriptome, and thereby the abundance of all transcripts can be quantified and presented as transcripts per million (TPM) after standardization through Cuffdiff software (9, 15).

### Microarray data acquisition

CHD-related microarray data were obtained from GEO (https://www.ncbi.nlm.nih.gov/geo), which is an available online genomic database that contains abundant gene expression profiles and relevant clinical information. We used the following search strategy: (1) (“coronary disease” [MeSH Terms] OR coronary heart disease [All Fields]) AND (“Expression profiling by array” [Study type] AND “Homo sapiens” [Top Organisms]) was adopted; (2) blood sample from all individuals; (3) each dataset contained more than six individuals. After rigorous screening, we selected one GPL570 dataset, GSE66360, which consists of 21 CHD samples and 22 healthy samples, as the test set along with our high-throughput sequencing data. Moreover, another GPL570 dataset GSE19339, one GPL9040 dataset GSE31568, and one GPL21825 dataset GSE160717, comprising 26 CHD samples and 26 healthy samples, were included as the validation sets to verify hub genes, miRNAs, and cicrRNAs involved in the pathogenesis of CHD, respectively. All data table header descriptions and series matrix files of gene expression profiles above were reprocessed and normalized by using the Robust Multiarray Average (RMA) method with R software 4.1.0 (10).

### Identification of differentially expressed genes

High-throughput sequencing directly obtains the sequence and quantity of all captured fragments through next-generation sequencing technology. However, microarray sequencing needs to synthesize the gene sequence of interest in advance and determine whether these genes are expressed through the fluorescent signal introduced by nucleic acid hybridization. The directness of high-throughput sequencing will inevitably make its overall accuracy and sequencing depth better than that of microarray sequencing. Considering the different methods and depths of data sequencing, the differentially expressed genes (DEGs) were screened using the following threshold criteria: (1) high-throughput sequencing data: log2 (fold change) > 4 or < −4 and adjusted *P-*value (*Q-*value) < 0.01; (2) microarray data: log2 (fold change) > 0.5 or < −0.5 and adjusted *P-*value (*Q-*value) < 0.05. The volcano plot and heatmaps of DEGs from each dataset were made with the limma package and pheatmap package of the R software. Additionally, system/organ-specific properties of all DEGs were identified by the online tool BioGPS (http://biogps.org/), which better indicated the distribution of genes in various tissues. Further online tool Venn Diagrams (http://www.bioinformatics.com.cn/static/others/jvenn/) were employed to exhibit DEGs shared by datasets.

### Functional and pathway enrichment analysis

The biological characteristics of all DEGs were annotated into molecular functions (MF), biological processes (BP), and cell components (CC) by using Gene Ontology (GO) enrichment analysis. In addition, the comprehensive gene interactions were presented through the Kyoto Encyclopedia of Genes and Genomes (KEGG) pathway analysis that could enrich DEGs into the molecular pathway. In our study, GO and KEGG pathway enrichment analyses were performed and visualized using the R software. The screening criteria were adjusted to *P* < 0.05.

Gene Set Enrichment Analysis (GSEA) software was initiated to carry out all genes to distinguish differential pathogenetic effects derived from the specific disease status. The screening criteria of significant gene sets were stated as follows: *P* < 5% and false discovery rate (FDR) < 25%.

### Immune infiltration analysis

Normalized expression profiling of the DEGs was imported to evaluate the relative content of 22 types of infiltrating immune cells by using the CIBERSORT (http://cibersort.stanford.edu/) to identify the immune characteristics of the dataset.

### Protein–protein interaction network construction

We constructed the PPI network of DEGs by searching the online tool Search Tool for the Retrieval of Interacting Genes/Proteins database (STRING; http://www.string-db.org/), which would predict and exhibit the interrelation of genes or proteins. Next, the PPI network was optimized with Cytoscape software, which could discover significant gene clusters through the module of Minimal Common Oncology Data Elements (MCODE) and identify hub genes *via* another plug-in Cytohubba.

### Construction of ceRNAs networks

We entered obtained hub genes into three miRNA databases, namely, miRDB, miRWalk, and targetScan, to predict corresponding target miRNAs and selected common miRNAs that were identified in at least three databases for downstream analysis. StarBase database (http://starbase.sysu.edu.cn/contact.php) was applied to identify circRNAs that targeted the miRNAs above. These miRNAs and circRNAs were intersected with CHD-related miRNAs, and circRNAs obtained from GEO and overlapped ones were constructed as the ceRNAs network, which was visualized by Cytoscape.

### Identification of potential drugs for CHD

Potential drugs related to selected hub genes were identified by using the Drug Gene Interaction Database (DGIdb; http://www.dgidb.org).

### Statistical analysis

All statistical data processing and analysis were performed with R software and SPSS Statistics 25.0. Comparisons of quantitative variables between the groups were performed by using Student's *t*-test. Pearson correlation analysis was used to elucidate the gene correlation. *P*-values < 0.05 were considered statistically significant.

## Results

### Identification of DEGs

**Figure 1:**
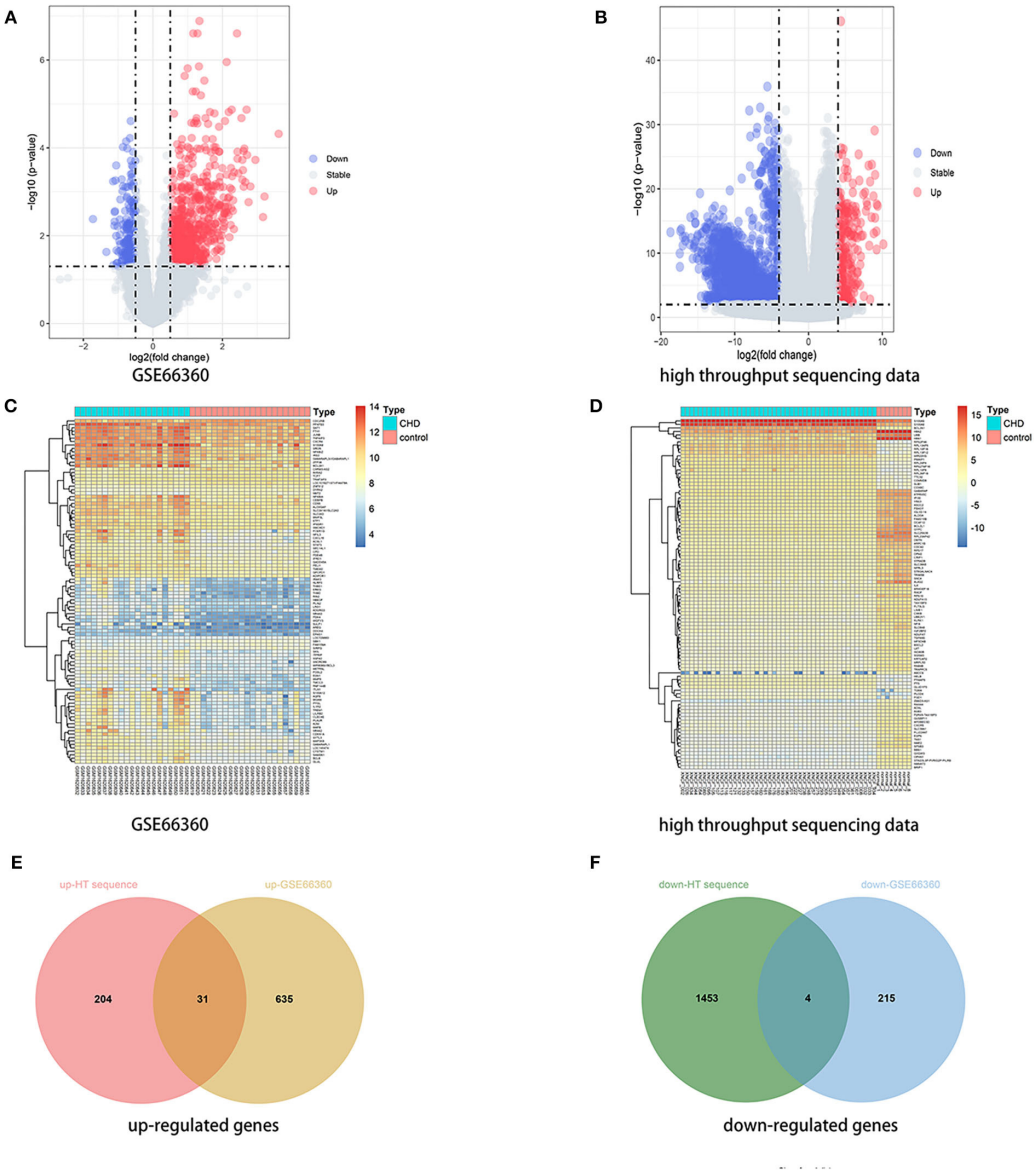
Identification of DEGs. **(A,B)** Volcano plot of DEGs between the CHD samples and the healthy samples from the GSE66360 dataset and high throughput sequencing dataset, respectively. Blue plots represent downregulated genes, gray plots represent nonsignificant genes, and red plots represent upregulated genes. **(C,D)** Heatmap of DEGs between the CHD samples and the healthy samples from the GSE66360 dataset and high throughput sequencing dataset, respectively. Blue rectangles represent low expression and red rectangles represent high expression. **(E,F)** Venn diagram of overlapped up-regulated and down-regulated DEGs from the two datasets, respectively.

A total of 1,692 and 885 DEGs were respectively identified from the high throughput sequencing dataset and GSE66360 based on the defined criteria, which were visualized using the volcano plots and the heatmaps . The high throughput sequencing dataset contains 235 up-regulated genes and 1,457 down-regulated genes, and GSE66360 contains 666 up-regulated genes and 219 down-regulated genes. The total overlap of the DEGs is presented in the Venn Diagram, including 31 up-regulated genes and 4 down-regulated genes (Figures 1E,F; Supplementary Table 1). The system/organ-specific properties of 35 DEGs were indicated by BioGPS (Supplementary Table 2), of which 19 DEGs are clustered in the hematologic/immune system (19/35, 54.28%).

### Enrichment analysis

**Figure 2:**
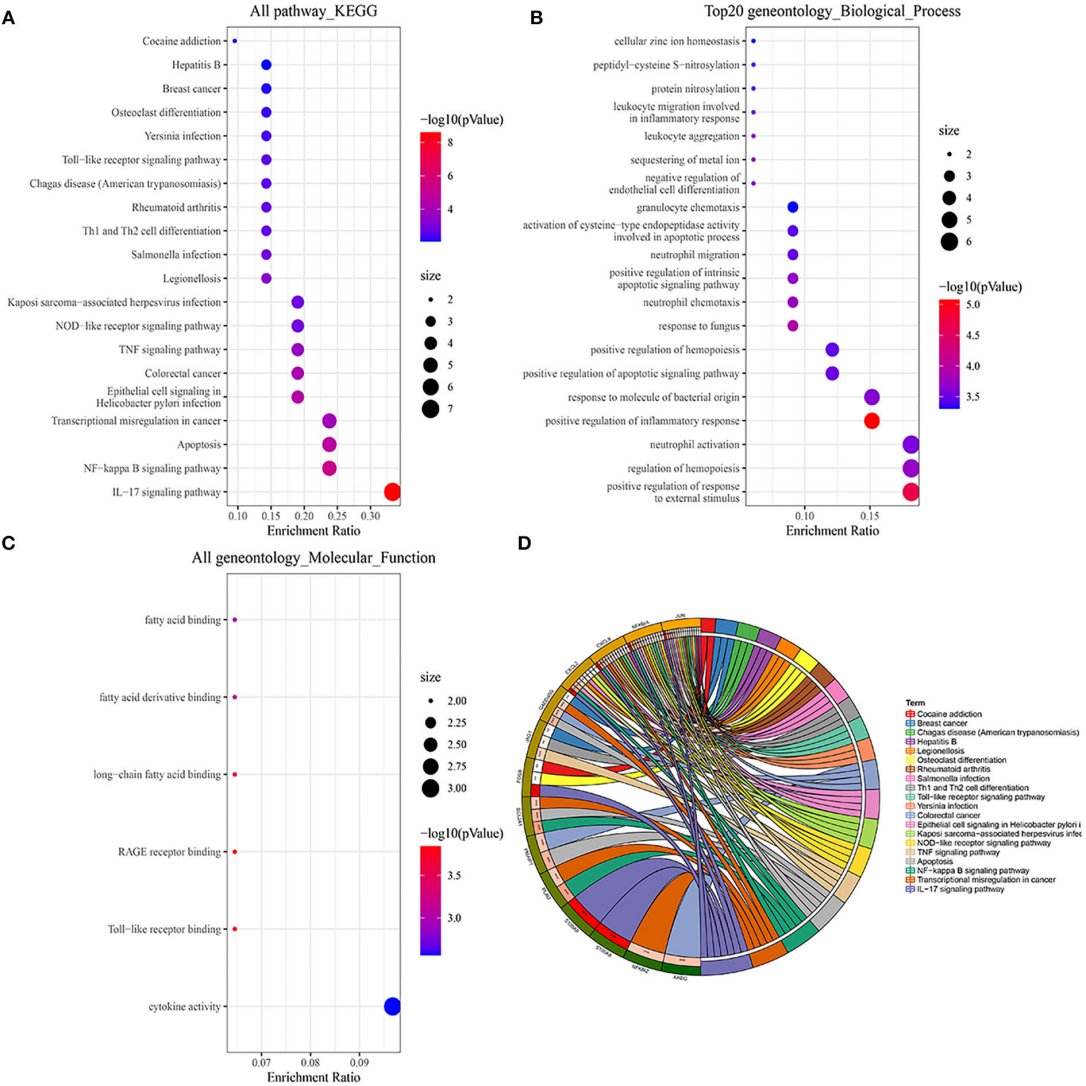
GO and KEGG pathway enrichment analysis of DEGs. **(A,D)** Bubble charts and chord plots both show KEGG pathway enrichment significance items of DEGs. **(B,C)** Two Bubble charts show GO enrichment significance items of DEGs in the two sections of BP and MF. In the Bubble charts, the Y-axis label represents enrichment significance terms and the X-axis label represents the gene ratio. In the Chord plot, DEGs are exhibited on the left side of the graph, and enrichment significance items are exhibited on the right side of the graph.

We first performed a KEGG pathway analysis of 35 overlapped DEGs that were mainly enriched in the exuberant immune response, including the IL-17 signaling pathway, NF-kappa B signaling pathway, TNF signaling pathway, and NOD-like receptor signaling pathway Figures 2A,D. In the GO enrichment analysis of these 35 DEGs, the significantly enriched categories for BP were positive regulation of response to external stimulus, regulation of hemopoiesis, neutrophil activation, and positive regulation of inflammatory response, and the prominent MF entries contained cytokine activity and Toll-like receptor binding (Figures 2B,C).

**Figure 3:**
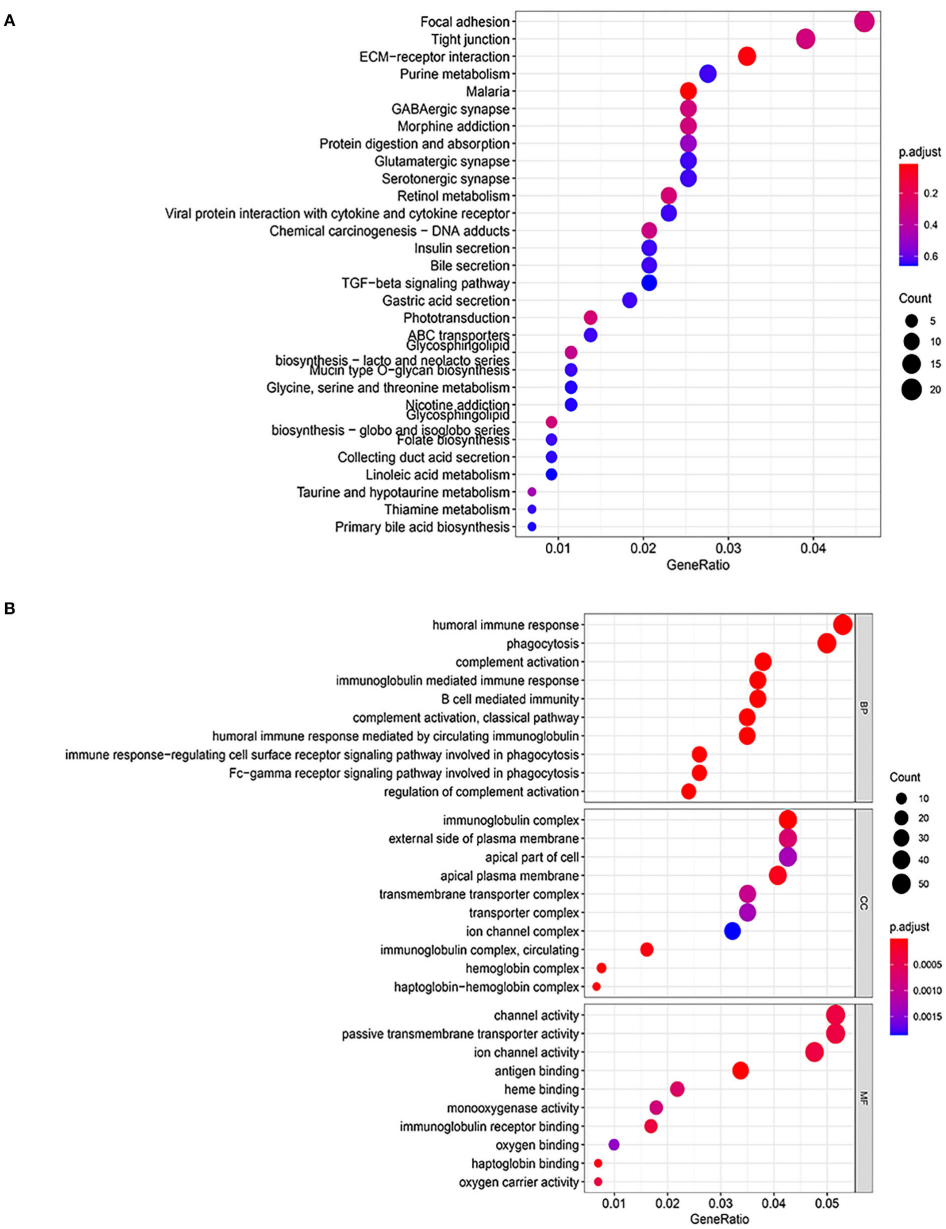
GO and KEGG pathway enrichment analysis of all DEGs of PCHD. **(A)** Bubble chart shows KEGG pathway enrichment significance items of DEGs. **(B)** Bubble chart shows GO pathway enrichment significance items of DEGs in three functional sections: biological processes (BP), cell composition (CC), and molecular function (MF). The Y-axis label represents enrichment significance terms and the X-axis label represents the gene ratio.

**Figure 4:**
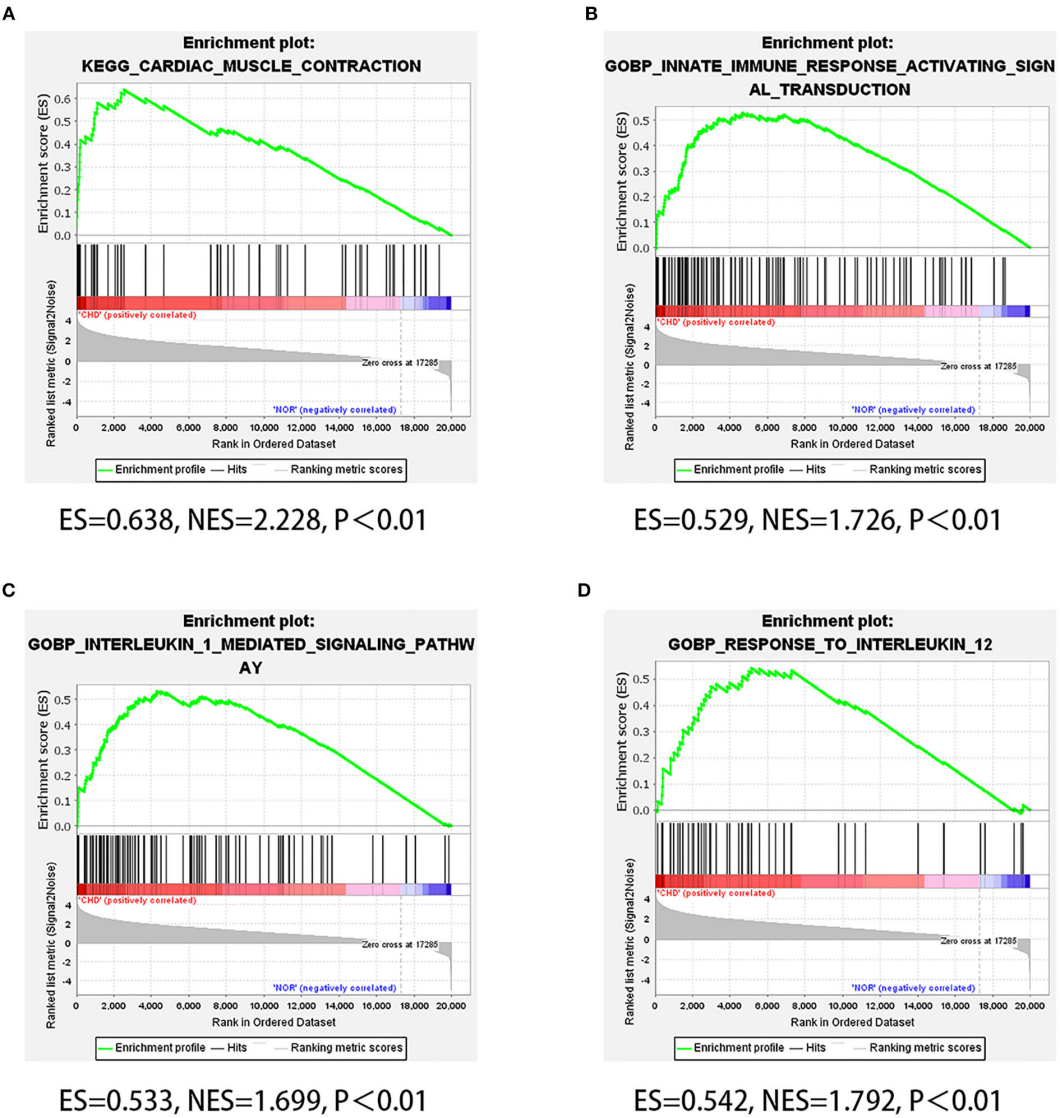
GSEA of high throughput sequencing dataset. **(A–D)** Four GSEA plots showing four significant gene sets of PCHD, which respectively are cardiac muscle contraction, innate immune response activating signal transduction, IL-1 mediated signaling pathway, and response to IL-12.

Considering the genetic attributes of PCHD, we further conducted a separate enrichment analysis on high-throughput sequencing dataset to obtain a preliminary understanding of its pathogenetic mechanism. KEGG pathway enrichment analysis of PCHD showed that all DEGs were mostly enriched in focal adhesion, tight junction, and ECM-receptor interaction (Figure 3A). The BP entries of GO enrichment included humoral immune response, phagocytosis, complement activation, immunoglobulin-mediated immune response, and B cell-mediated immunity. Regarding CC, the significant categories were the immunoglobulin complex and the external side of the plasma membrane. For MF, enriched terms were channel activity and passive transmembrane transporter activity (Figure 3B). Additionally, GSEA was carried out to indicate that the most significant-enriched gene sets, namely, cardiac muscle contraction, innate immune response activating signal transduction, IL-1 mediated signaling pathway, and response to IL-12, positively correlated with PCHD (Figure 4).

### Immune infiltration characteristics of high throughput sequencing dataset

**Figure 5:**
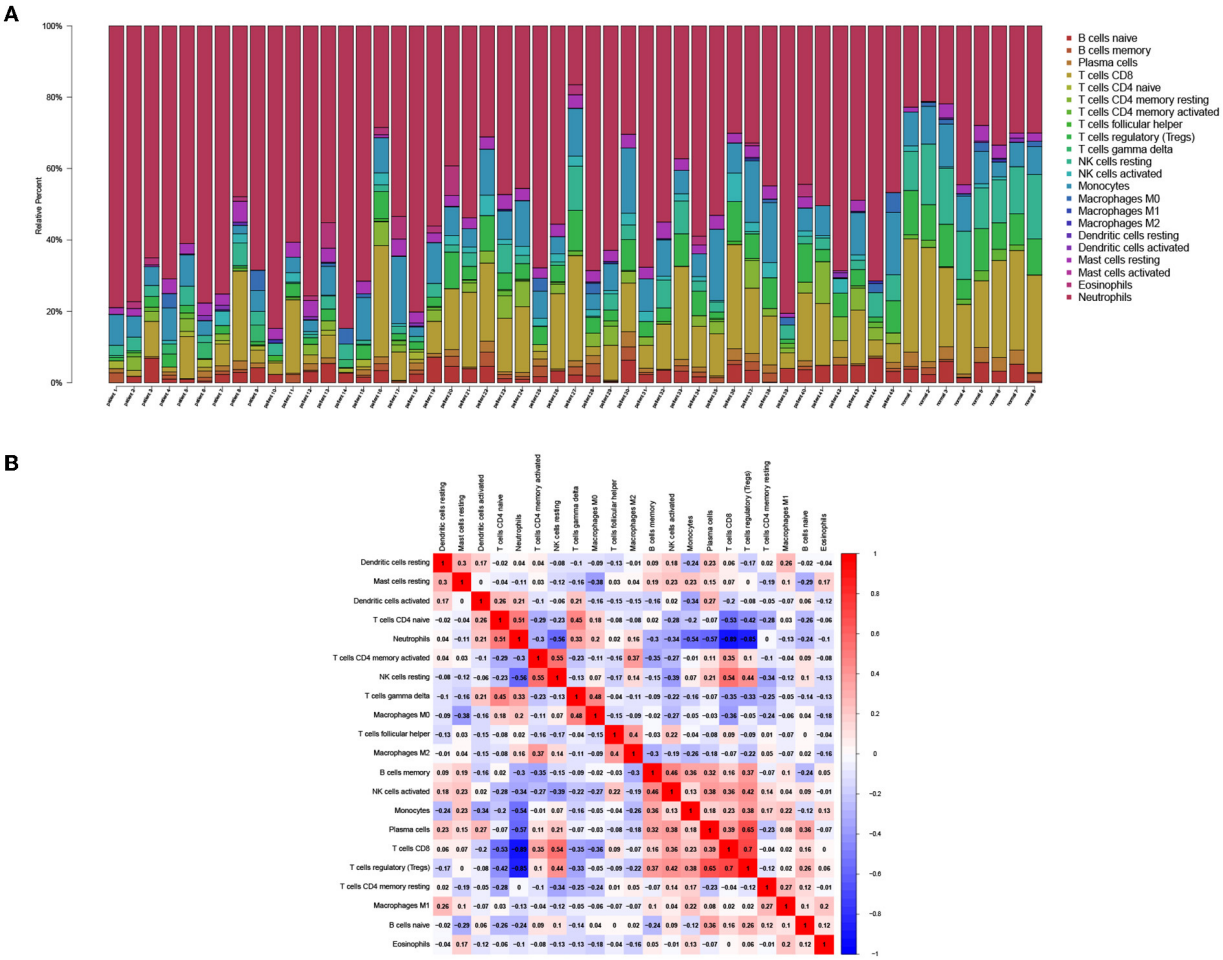
Immune infiltration analysis of DEGs from high throughput sequencing dataset. **(A)** Bar charts showing the proportions of immune cell subsets. **(B)** Correlation heatmap showing the association of various immune cell subsets.

To demonstrate the distribution of immune cells in PCHD, we found that resting memory CD4 T cells, resting mast cells, eosinophils, and neutrophils were more abundant; however resting NK cells, M2 macrophages, memory B cells, CD8 T cells, naïve CD4 T cells were relatively scarce in patients with PCHD by using CIBERSORT algorithm Figure 5A. The proportion of neutrophils was negatively associated with that of CD8 T cells and T regulatory (Treg) cells (Figure 5B).

### PPI network analysis

**Figure 6:**
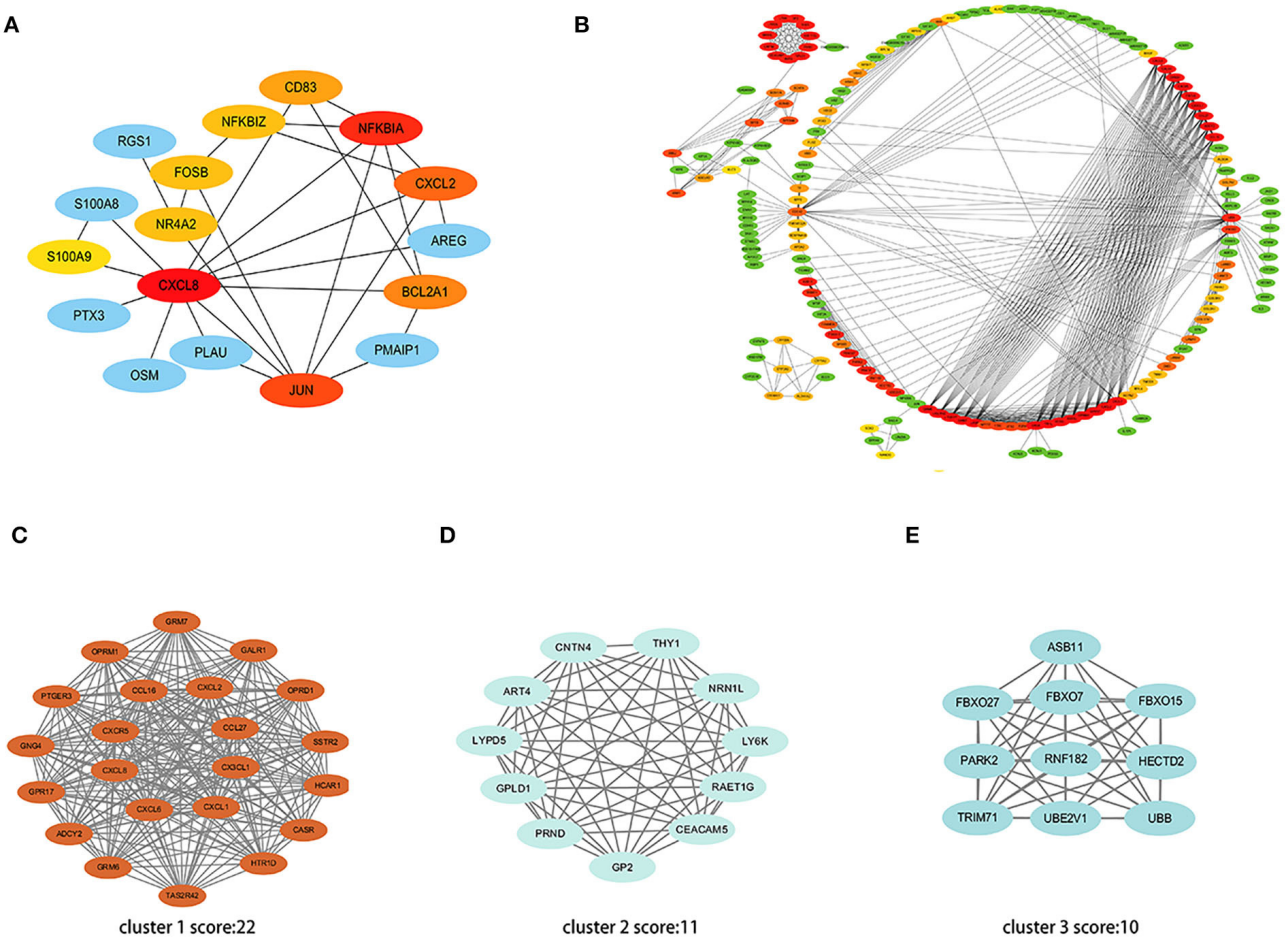
PPI network of DEGs. **(A)** The interaction network shows the important genes and their interactions among 35 co-expressed DEGs. **(B)** The interaction network shows the important genes and their interactions in the high throughput sequencing dataset. **(C–E)** Three most significant clusters of the high throughput sequencing dataset. Each node represents a protein, while each edge represents one protein-protein association.

A total of 35 co-expressed DEGs were entered into the online tool STRING and constructed as a PPI interaction network with 17 nodes and 28 edges under the condition of scattered nodes deletion (Figure 6A). Next, cytohubba was applied to identify 10 hub genes presented with red and yellow color.

Further, we put all the DEGs of the high throughput sequencing dataset into STRING and screened the top 100 hub genes using algorithms of cytohubba (Figure 6B). The MCODE plugin was used to verify the most significant cluster 1 consisting of 22 nodes (MCODE score: 22), followed by cluster 2 (MCODE score: 11) and cluster 3 (MCODE score: 10) (Figures 6C–E).

### Verification of 10 hub genes in GSE19339 dataset

**Figure 7:**
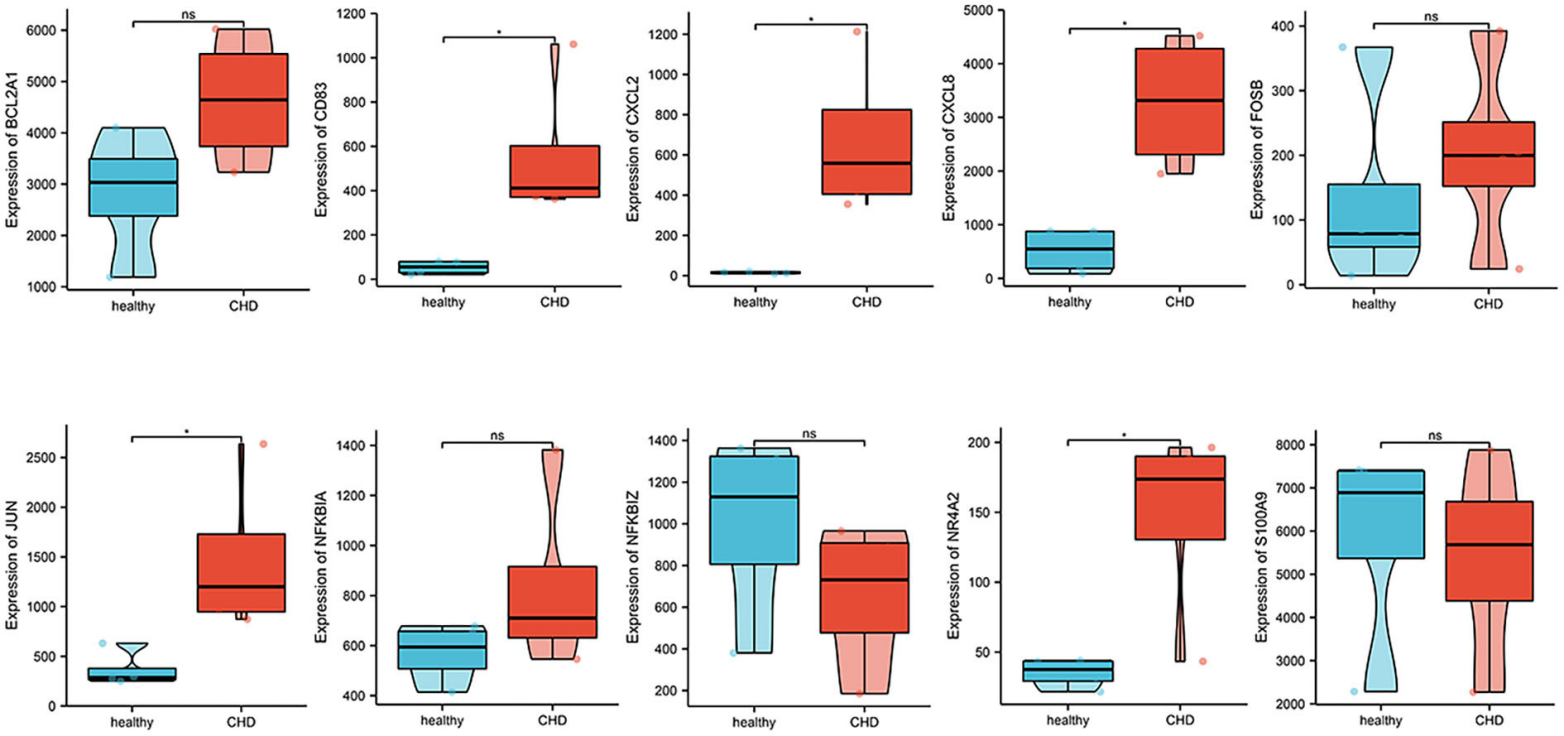
The validation of ten hub genes in the GSE19339 dataset.

Expression profiles of 10 hub genes were extracted from GSE19339 and compared between the two groups. We observed that the levels of *CD83, CXCL2, CXCL8, JUN*, and *NR4A2* were significantly increased in the CHD group (*P* < 0.05) (Figure 7).

### Target ncRNAs prediction and network construction

**Figure 8:**
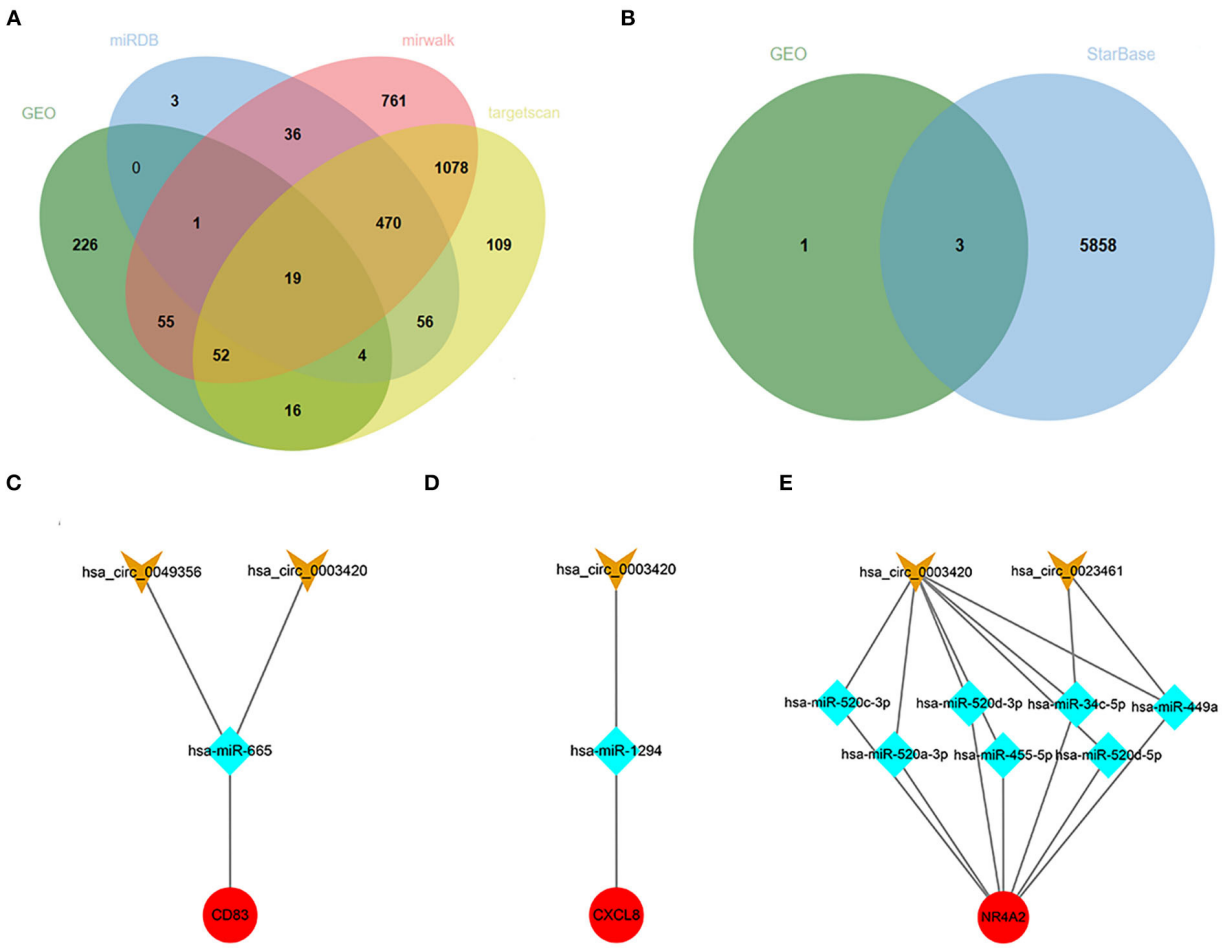
Target ncRNAs Prediction and Network Construction. **(A)** Venn diagram of overlapped miRNAs from the GSE31568 dataset and online miRNA databases. **(B)** Venn diagram of overlapped circRNAs from GSE160717 dataset and online circRNAs databases. **(C–E)** Three ceRNA network of *CD83, CXCL8*, and *NR4A2*. Red nodes represent hub genes, blue nodes represent miRNAs, and orange nodes represent circRNAs.

A total of 201 hub-gene-related miRNAs were obtained based on three miRNA databases, and we screened out 397 CHD-related miRNAs by differential genes expression analysis in the GSE31568 dataset. Finally, 19 intersected miRNAs and their corresponding mRNAs were exhibited in the Venn diagram and interaction networks figure (Figure 8A).

Using the same method as above to predict circRNAs, we successively searched the StarBase database, analyzed data from GSE160717, and took intersections as target circRNAs. Finally, three ceRNA networks of *CD83, CXCL8*, and *NR4A2* were constructed, and they might be potential RNA regulatory pathways to regulate PCHD progression (Figures 8B–E).

### Identification of the potential drugs

**Figure 9:**
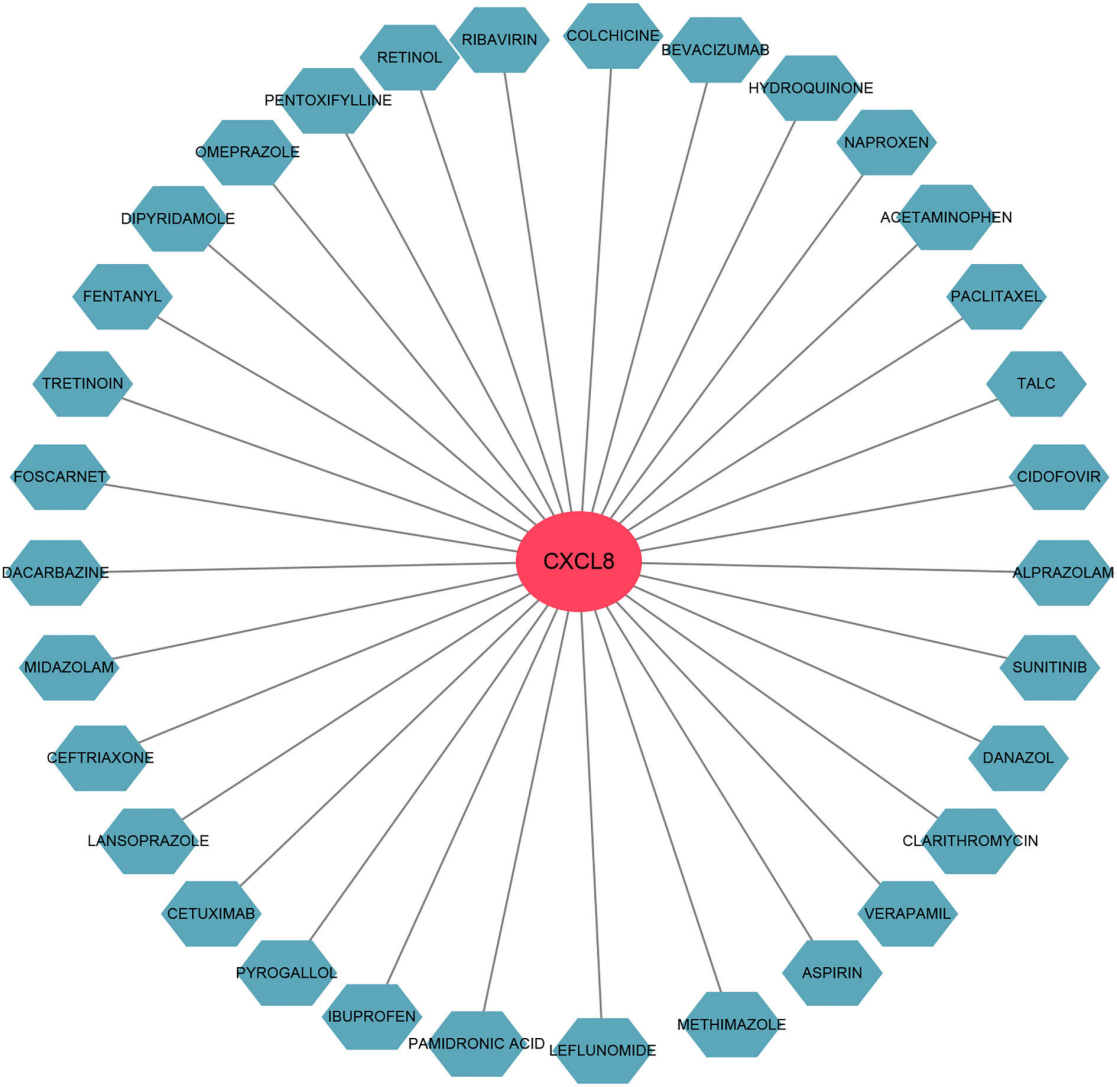
The gene-drug interaction network. The red node represents the hub gene and ocean blue nodes represent drugs.

We used the DGIdb database to predict the potential drugs that might reverse the detrimental effects of DEGs in PCHD. As a result, *CXCL8* interacted most with drugs, namely, cetuximab, acetaminophen, bevacizumab, leflunomide, colchicine, paclitaxel, etc., (Figure 9).

## Discussion

Approximately 10% of the young population under the age of 45 develop CHD, which contributes greatly to the current global health and economic burden (11). The chronic and systemic inflammations perpetuate this disease and are positively correlated with the subsequent major adverse cardiovascular events (3, 16). Even if patients with CHD receive optimal medical treatment or invasive percutaneous coronary intervention (PCI), a substantial portion of them still suffer from recurrent myocardial infarction or elective revascularization (3, 5). Recent studies demonstrated that the onset and progression of CHD could be intervened by polygenetic variants that were involved in multiple biological pathways, including the regulation of hypertension, dyslipidemia, hyperglycemia, vascular remodeling, and inflammation (17–19). It is firmly believed that patients with PCHD with no clear traditional cardiovascular risk factors might be more likely to be modulated by inherited risk factors (2, 20). This indicates that the identification of novel genetic biomarkers with maximal specificity and sensitivity is rationally demanded to prevent and diagnose PCHD earlier.

In the present study, we recruited 45 Chinese Han patients with PCHD as the investigated population and draw their peripheral blood samples for high throughput sequencing. Since no available sequencing data related to PCHD have been reported before, we retrieved CHD-related data as a reference from the online public genomic database. Through data processing and differential gene expression analysis, a set of whole blood gene expression changes specific to those with PCHD were compared to healthy individuals, suggesting a direct correlation between myocardial injury and blood gene expression (12). Moreover, we identified the total overlap of 35 DEGs from two datasets, including 19 hematologic/immune system-specific expressed genes. These findings were consistent with a recently published study focusing on unstable angina patients, which preliminarily indicated that immune dysfunction was involved in the pathophysiological processes of this disease (9). GO and KEGG enrichment analysis of 35 overlapped DEGs indicated that the immune responses, such as neutrophil activation, IL-17 signaling pathway, NF-kappa B signaling pathway, TNF signaling pathway, and NOD-like receptor signaling pathway, were exuberant in patients with CHD compared to healthy individuals. Considering the genetic attributes of PCHD, we further conducted the enrichment analysis of all PCHD-related DEGs, which also were mostly enriched in excessive immune activation pathways and abundant signal transduction. Additionally, we performed a GSEA algorithm that could identify differential enrichment functions based on the overall expression trend of all genes in the PCHD dataset. The results demonstrated that most of the genes in subjects with PCHD were mainly clustered in the cardiac muscle contraction, innate immune response activating signal transduction, and response to IL-12 and IL-1 mediated-signaling pathway, suggesting that immune dysfunctions have been again confirmed as the essential mechanism contributing to PCHD (21). The proportions of 22 subsets of infiltrating immune cells from PCHD transcriptomes were calculated by conducting a state-of-the-art deconvolution CIBERSORT algorithm. We found that higher proportions of neutrophils and eosinophils were associated with PCHD, which was in line with the results of GO and KEGG enrichment analysis in this study. Abundant evidence has implicated the essential roles of neutrophils that release a series of inflammatory cytokines in plaque progression (3, 22). We also found that M2 macrophages, CD8 T cells, and Treg cells were relatively scarce in patients with PCHD. M2 macrophages and Treg cells are however pivotal anti-inflammatory ingredients that could render plaque stabilization by secreting anti-atherogenic cytokines (16, 23, 24). It could be interpreted that PCHD was prone to occur when protective immune cells were dampened. All findings above in our study are consistent with the progression of coronary atherosclerotic plaques that are composed of a thin fibrous cap and large necrotic cores involving activated immune cells and inflammatory cytokines (23). We have identified important inflammatory pathways and immune cell subsets in patients with PCHD at the transcriptome and cell level through integrated bioinformatics methods, which could provide a more detailed understanding of disease pathogenesis.

Ten hub genes were recognized by constructing the PPI network and analyzing it by Cytoscape. Through verifying the expression levels of these hub genes in 53 recruited participants and another CHD dataset, we identified five critical genes that included *CD83, CXCL2, JUN, CXCL8*, and *NR4A2*. Various studies demonstrated that the gene expression could generally be down-regulated and even silenced by combining with miRNAs; however, upstream circRNAs could intervene with miRNAs response elements (10). This regulatory interaction among RNAs is called a ceRNA network. We thus crossed the GEO, miRNA databases, and StarBase database to obtain three ceRNA networks of *CD83, CXCL8*, and *NR4A2* to elucidate the pathogenesis of CHD. *CD83*, a member of the immunoglobulin superfamily, is a characteristic marker on the surface of dendritic cells. A continuous high expression of *CD83* represents abundant dendritic cells that participate in antigen presentation and lymphocyte activation (25). IL-12, TGF-β, and IL-1β released by dendritic cells promote the maturation of helper T 1 cells to induce inflammatory cascades (16, 25). Previous studies claimed that activated dendritic cells migrated into atherosclerotic plaque and accelerated its progression (23, 25). *CXCL8* (also called *IL-8*), a contributor to the local inflammatory response, has been considered to chemoattract neutrophils that move toward progressive plaque and produce excessive inflammatory cytokines (26). *CXCL8* also is confirmed as a center molecule involved in the IL-17 signaling pathway, NF-kappa B signaling pathway, and NOD-like receptor signaling pathway, which is consistent with GO and KEGG enrichment analysis in our study (26–28). An up-regulated *CXCL8* gene is thus associated with a high incidence of PCHD. *NR4A2* is a member of the NR4A orphan nuclear receptor family, which encodes a zinc finger protein that can bind to DNA. *NR4A2* is considered an adaptive response gene that can be activated in response to various stresses (29). Mutations in this gene have been associated with disorders related to dopaminergic dysfunction, such as Parkinson's disease and manic depression (30). In addition, *NR4A2* is also up-regulated by the upstream molecule TNFα in synovial tissue, contributing to cartilage destruction and ultimately rheumatoid arthritis. A recent study revealed that *NR4A2* was upregulated in mice with ischemia-induced myocardial damage, and the overexpression of *NR4A2* would improve myocardial apoptosis (31). Up-regulation of *NR4A2* might be therefore self-protective with progressive pro-inflammatory infiltration of patients with PCHD in our study. Our realization of these three ceRNA networks regarding inflammatory activation could be further rationally explored as potential diagnostic biomarkers for PCHD.

To predict the promising effective therapy for PCHD, we used the DGIdb database to discover promising therapeutic agents that might reverse the detrimental effects of *CD83, CXCL8*, and *NR4A2*. The results indicated that only *CXCL8* could be interfered with by multiple chemical compounds mainly consisting of biological monoclonal antibodies and non-steroidal and anti-inflammatory drugs. The CANTOS trial suggested the efficacy of canakinumab targeting IL-1β in improving the prognosis of patients with CHD (32). Subsequent evidence has supported the idea that multiple anti-inflammatory manipulations exert their cardiovascular benefits with controllable adverse reactions through the resolution of inflammation (4). Among our predicted drugs, colchicine targeting *CXCL8* has been confirmed by COLCOT, LoDoCo-2, COPS, and COLCHICINE-PCI trials to show significant clinical benefits, indicating that other predicted agents have the potential to be administrated in future individualized treatment of PCHD.

There were several limitations in this study. Various investigations demonstrated that the whole blood transcriptome could be viewed as an accessible window to the multiorgan transcriptome (33), and our study was the first one to explore differences in gene expression of Chinese Han patients with PCHD. Therefore, a comparison of transcriptome differences in blood samples from individuals with and without PCHD in our study exhibited distinct differences in gene regulation changes related to PCHD from the genome-wide perspective. However, multi-gene interactions and their complex functions require cellular and animal experiments for verification. Second, the blood samples were drawn from all participants before or after medication and PCI; we were not yet sure about the possible effects of treatments on gene transcription and cell subsets. Third, the cohorts included in this study contained multiple ethnicities, which might be an important risk factor involved in the pathogenesis of CHD (34). Further investigations into more Chinese individuals are needed for the confirmation of our results to exclude potential racial bias.

## Conclusion

We identified three hematologic/immune system-specific expressed genes, *CD83, CXCL8*, and *NR4A2*, that could control disease progression through the ceRNA network in PCHD. Although the uncertainties warrant further evidence, these three genes might be potential biomarkers for clinical applications regarding early diagnosis, targeted therapy, and prognosis evaluation of PCHD.

## Data availability statement

The datasets presented in this study can be found in online repositories. The names of the repository/repositories and accession number(s) can be found in the article/Supplementary material.

## Ethics statement

The studies involving human participants were reviewed and approved by the Ethics Committee of the Chinese PLA General Hospital. The patients/participants provided their written informed consent to participate in this study.

## Author contributions

HW and JS: data analysis, validation and interpretation, figures production, and writing. XLu and XLi: statistical analysis. MJ, ZL, YZ, JZ, LL, LW, and QX: clinical sample collection and partial data analysis and validation. YC and RZ: study design, data interpretation and validation, and writing and manuscript revision. All authors contributed to the article and approved the submitted version.

## Funding

This work was supported by The National Key Research and Development Program of China (Grant Nos. 2021YFC2701700 and 2021YFC2701703).

## Conflict of interest

The authors declare that the research was conducted in the absence of any commercial or financial relationships that could be construed as a potential conflict of interest.

## Publisher's note

All claims expressed in this article are solely those of the authors and do not necessarily represent those of their affiliated organizations, or those of the publisher, the editors and the reviewers. Any product that may be evaluated in this article, or claim that may be made by its manufacturer, is not guaranteed or endorsed by the publisher.
