# Supplementary material for: Potential of immune-related genes as promising biomarkers for premature coronary heart disease through high throughput sequencing and integrated bioinformatics analysis
Source: Front Cardiovasc Med. 2022 Aug 26;9:893502. doi: 10.3389/fcvm.2022.893502 (PMC9458892; doi:10.3389/fcvm.2022.893502)
Supplement: Supplementary file 1 [file Table_1.DOCX]

**Supplementary Table 1 Differentially expressed genes of CHD**

| **Gene** | **Adjusted *P* value** | | | **Fold Change** | | **Description** | **Genome Location** |
| --- | --- | --- | --- | --- | --- | --- | --- |
|  | **HT sequencing** | **GSE66360** | **HT sequencing** | | **GSE66360** |  |  |
| **Up-regulated** | |  |  | |  |  |  |
| AREG | 3.62E-11 | 0.000105 | 6.678 | | 1.362 | amphiregulin | chr4:74445136-74455005 |
| B3GNT5 | 6.13E-11 | 0.032163 | 4.112 | | 0.617 | beta-1,3-N-acetylglucosaminyltransferase 5 | chr3:183253253-183298504 |
| BCL2A1 | 1.54E-22 | 0.000250 | 5.301 | | 1.785 | BCL2 related protein A1 | chr15:79960892-79971196 |
| C15orf48 | 2.67E-08 | 0.008529 | 4.509 | | 1.867 | chromosome 15 open reading frame 48 | chr15:45430579-45448761 |
| CD83 | 1.86E-12 | 0.000582 | 4.222 | | 1.541 | CD83 molecule | chr6:14117256-14136918 |
| CXCL2 | 2.67E-11 | 0.005040 | 9.225 | | 2.051 | C-X-C motif chemokine ligand 2 | chr4:74097040-74099196 |
| CXCL8 | 2.47E-18 | 0.021745 | 9.233 | | 1.233 | C-X-C motif chemokine ligand 8 | chr4:73740541-73743716 |
| FOSB | 2.37E-09 | 0.007331 | 4.377 | | 1.514 | FosB proto-oncogene, AP-1 transcription factor subunit | chr19:45467995-45475179 |
| G0S2 | 4.39E-18 | 0.012402 | 9.368 | | 1.364 | G0/G1 switch 2 | chr1:209675412-209676390 |
| GADD45G | 4.99E-17 | 0.010616 | 5.322 | | 0.513 | growth arrest and DNA damage inducible gamma | chr9:89605012-89606555 |
| GJB6 | 2.25E-05 | 0.039972 | 6.222 | | 1.045 | gap junction protein beta 6 | chr13:20221962-20232365 |
| GK3P | 1.82E-08 | 0.027831 | 4.730 | | 0.978 | glycerol kinase 3 pseudogene | chr4:165277812-165279679 |
| ID1 | 1.70E-13 | 0.007109 | 5.726 | | 0.815 | inhibitor of DNA binding 1 | chr20:31605283-31606515 |
| JAG1 | 1.63E-18 | 0.000711 | 4.271 | | 1.049 | jagged canonical Notch ligand 1 | chr20:10637684-10673999 |
| JUN | 2.99E-10 | 0.000146 | 4.004 | | 0.772 | Jun proto-oncogene, AP-1 transcription factor subunit | chr1:58776845-58784048 |
| KLHL15 | 7.60E-17 | 0.032453 | 4.603 | | 0.598 | kelch like family member 15 | chrX:23983720-24027186 |
| MED14OS | 1.55E-17 | 0.036961 | 4.671 | | 0.509 | MED14 opposite strand | chrX:40735400-40738701 |
| NFKBIA | 1.04E-14 | 9.57E-05 | 4.036 | | 1.495 | NFKB inhibitor alpha | chr14:35401513-35404749 |
| NFKBIZ | 1.05E-17 | 0.000122 | 4.410 | | 1.162 | NFKB inhibitor zeta | chr3:101827991-101861022 |
| NR4A2 | 2.45E-18 | 2.10E-05 | 5.113 | | 2.546 | nuclear receptor subfamily 4 group A member 2 | chr2:156324437-156342348 |
| OSM | 7.03E-13 | 0.003524 | 4.895 | | 1.014 | oncostatin M | chr22:30262829-30266851 |
| PLAU | 1.24E-07 | 0.000288 | 4.813 | | 1.997 | plasminogen activator, urokinase | chr10:73909177-73917496 |
| PLK2 | 2.79E-17 | 0.009934 | 6.374 | | 0.863 | polo like kinase 2 | chr5:58453982-58460139 |
| PMAIP1 | 9.07E-24 | 0.002828 | 4.907 | | 1.079 | phorbol-12-myristate-13-acetate-induced protein 1 | chr18:59899996-59904305 |
| PTS | 4.04E-26 | 0.023102 | 4.589 | | 0.566 | 6-pyruvoyltetrahydropterin synthase | chr11:112226367-112269955 |
| PTX3 | 8.08E-16 | 0.003766 | 4.887 | | 3.167 | pentraxin 3 | chr3:157436850-157443633 |
| RGS1 | 1.04E-18 | 0.003447 | 7.863 | | 0.897 | regulator of G protein signaling 1 | chr1:192575763-192580024 |
| S100A8 | 3.45E-20 | 0.000454 | 6.000 | | 1.930 | S100 calcium binding protein A8 | chr1:153390032-153391073 |
| S100A9 | 8.33E-47 | 0.001343 | 4.389 | | 2.032 | S100 calcium binding protein A9 | chr1:153357854-153361023 |
| SIGLEC5 | 1.16E-05 | 0.025422 | 4.746 | | 1.354 | sialic acid binding Ig like lectin 5 | chr19:51610960-51646889 |
| TULP2 | 6.87E-12 | 0.008942 | 8.226 | | 1.665 | TUB like protein 2 | chr19:48880967-48898744 |
|  |  |  |  | |  |  |  |
| **Down-regulated** | |  |  | |  |  |  |
| ANK1 | 4.14E-18 | 0.004482 | -4.980 | | -0.634 | ankyrin 1 | chr8:41653220-41896762 |
| SLITRK5 | 5.28E-05 | 0.036419 | -4.722 | | -0.546 | SLIT and NTRK like family member 5 | chr13:87671371-87696272 |
| TSPAN5 | 8.38E-21 | 0.014762 | -4.489 | | -0.681 | tetraspanin 5 | chr4:98470367-98658611 |
| ZNF578 | 2.82E-05 | 0.021393 | -4.842 | | -0.731 | zinc finger protein 578 | chr19:52453553-52516882 |
