# Supplementary material for: Potential of immune-related genes as promising biomarkers for premature coronary heart disease through high throughput sequencing and integrated bioinformatics analysis
Source: Front Cardiovasc Med. 2022 Aug 26;9:893502. doi: 10.3389/fcvm.2022.893502 (PMC9458892; doi:10.3389/fcvm.2022.893502)
Supplement: Supplementary file 2 [file Table_2.DOCX]

**Supplementary Table 2 System/organ-specific expressed genes identified by BioGPS**

| **System/Organ** | **Genes** | **Counts** |
| --- | --- | --- |
| Hematologic/Immune | S100A9, PMAIP1, BCL2A1, TSPAN5, CXCL8, NR4A2, ANK1, NFKBIZ, MED14OS, KLHL15, NFKBIA, OSM, CD83, CXCL2, GK3P, C15orf48, SIGLEC5, GJB6, ZNF578 | 19 |
| Genital | PTX3, ID1, TULP2, AREG, B3GNT5, JUN, PLAU, SLITRK5 | 8 |
| Respiratory | PTS, JAG1 | 2 |
| Nervous | RGS1, PLK2 | 2 |
| Circulatory | GADD45G | 1 |
| Breast | FOSB | 1 |
| Adipose | G0S2 | 1 |
| Tongue | S100A8 | 1 |
